# SpyBite-Assisted Biliary Cannulation Facilitating Endoscopic Retrograde Cholangiopancreatography in the Presence of Intradiverticular Papilla

**DOI:** 10.14309/crj.0000000000001543

**Published:** 2024-11-06

**Authors:** Juan Sebastián Frías-Ordoñez, Martín Alonso Garzón Olarte, Geovanny Hernández-Cely

**Affiliations:** 1 Gastroenterology and Digestive Endoscopy, National University of Colombia, Bogotá, Colombia; 2 Gastroenterology and Hepatology, Cardioinfantil-La Cardio Foundation, Bogotá, Colombia

**Keywords:** cholangiopancreatography, endoscopic retrograde, biliary tract diseases, periampullary diverticula, SpyBite, difficult biliary cannulation

## Abstract

Biliary cannulation in the context of intradiverticular papilla (IDP) during endoscopic retrograde cholangiopancreatography (ERCP) remains a challenge even for experts. A 71-year-old woman with choledocholithiasis failed extrainstitutional ERCP because of the presence of IDP. A new ERCP was performed, identifying papillary orifice at the edge of the diverticulum, biliary cannulation by means of SpyBite and sphincterotome, visualizing intra and extrahepatic bile duct dilatation, proceeding to large balloon bilioplasty with extraction of biliary sludge, with optimal results and without complications. This strategy was effective and safe. It could be recommended for difficult cannulation in the presence of IDP.

## INTRODUCTION

Intradiverticular papilla (IDP) presentation can hinder biliary cannulation during endoscopic retrograde cholangiopancreatography (ERCP). It occurs in up to occurs in up to 32% of cases and requires specialized techniques by experienced endoscopists.^1^ Its prevalence increases with age, being 65% in older adults.^2^ In comparison with patients without diverticulum, a lower cannulation rate has been demonstrated.^3^ The Li-Tanaka classification should be considered when approaching it, those cases with the papilla completely located in the diverticulum (type I) have the lowest cannulation rate (23.1%), compared with those cases with papilla located in the margin of the diverticulum (type II), or outside the margin of the diverticulum (III), with cannulation success rates of 99.4% and 99.3%, respectively.^4^

There are different techniques to facilitate biliary cannulation in the presence of IDP, most of which have been described in case reports and case series. Some of these techniques include endoscopic ultrasound (EUS) or percutaneous-assisted rendezvous technique, endoclip-assisted cannulation with double wire-guide, and the two-devices-in-one-channel method, among others.^5–7^ The use of SpyBite forceps (Boston Scientific, Natick, MA) was described by Levenick et al in 2014.^8^ in a case of biliary leak on the eighth postoperative day after open cholecystectomy, and this technique allows changing the anatomical position of the papilla, reducing redundant tissue, maintaining the ampullary position, and gaining access. A case is described below in which ERCP was successfully performed using SpyBite forceps in a patient with IDP with Li-Tanaka type II classification. The results found in the present case confirm its efficacy and safety in these cases of difficult biliary cannulation.

## CASE REPORT

A 71-year-old woman with a history of open cholecystectomy (2010) is referred for chronic recurrent biliary colic and documentation of choledocholithiasis. In another institution, it was identified proximal dilatation of the common bile duct with distal stenosis of unclear cause by cholangioresonance imaging. An ERCP was attempted and was not successful because of an IDP type II. The patient was referred to our institution.

**Figure 1.**
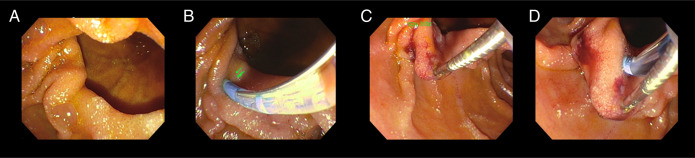
Biliary cannulation in IDP type II using SpyBite and TRUEtome sphincterotome (Boston Scientific, Natick, MA). (A) IDP is observed without being able to adequately identify the papillary orifice. (B) Cannulation is attempted with papillotome without being able to visualize papillary orifice. (C) By means of SpyBite, the diverticular edge is taken to be able to confront the papillary lumen. (D) Using a TRUEtome sphincterotome (Boston Scientific, Natick, MA), placing the tip at 11 o'clock and 0.025 guide, access to the biliary duct is achieved with difficulty. IDP, intradiverticular papilla.

In the ERCP, IDP was identified with papillary orifice at the edge of the diverticulum (Li-Tanaka type II), without being possible its cannulation by conventional methods, so we proceeded to cannulate by means of SpyBite and TRUEtome sphincterotome (Boston Scientific, Natick, MA) (Figure 1).

**Figure 2.**
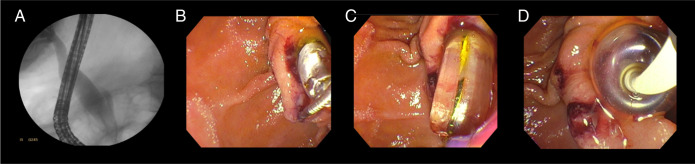
Identification of bile duct involvement and bilioplasty with biliary sludge extraction. (A) Contrast injection showing a dilated extrahepatic bile duct, slightly dilated intrahepatic bile duct, and absence of filling defects. (B) Bilioplasty with Hurricane (Boston Scientific, Natick, MA) at 6 mm, no sphincterotomy is performed because no papillary safety edges are observed. (C) Bilioplasty is performed with a large balloon at 10 mm. (D) Fogarty balloon is advanced with drainage of biliary sludge and abundant dark bile. Source: authors.

Once the biliary tract was cannulated, the biliary tract compromise was identified, and large balloon bilioplasty with biliary sludge extraction was performed (Figure 2). Achieving optimal results, the patient remained under intrahospital surveillance for 12 hours to evaluate oral tolerance and absence of complications. The patient remained asymptomatic in her outpatient follow-up at the third month.

## DISCUSSION

ERCP in patients with IDP requires specialized methods and experienced personnel, and the possibility of cannulation and successful completion of endoscopic therapy will depend on several factors among which are (i) the position of the papilla according to the diverticulum; (ii) gallstone formation in the presence of IDP; (iii) the experience of the endoscopist; and (iv) the available supplies.

ERCP cannulation in IDP is more difficult in cases where the papilla is located at 1 o'clock compared with other papilla locations, and the cannulation rate in IDP can range from 61% to 95.4% and may require a combination of methods to achieve cannulation.^4,9^ In the present case, traction with SpyBite was required for better visualization of the papillary orifice after failure with conventional methods, achieving its visualization at 11 o'clock, being a usual cannulation position for the endoscopist.

Patients with IDP are 6 times more likely to have retained lithiasis compared with those without IDP.^10,11^ Some factors associated with its plausibility include sphincter of Oddi dysfunction, sphincter spasm caused by the diverticulum, and increased biliary tract or diverticulum pressure, leading to bile stasis by distal compression of the common bile duct.^11^ The patient in the present case was characterized by long-standing biliary disease and a history of cholecystectomy, and although no gallstones were documented, she had a dilated bile duct in relation to biliary sludge and bile retention.

Finally, the method of choice in case of IDP will depend on the preference of the endoscopist, the supplies available in the institution, and the conditions of the patient. The main objective of the different methods will be to approximate the papilla in a better position and angle suitable for cannulation. The current description in conjunction with the previous report by Levenick et al^8^ positions the use of SpyBite as an available technique, useful and safe in the successful cannulation of patients with IDP. However, there are pitfalls, such as the cost and disponibility across the centers. Also, there are no randomized clinical trials that dictate the adequate technique to follow. Prospective randomized trials and comparative efficacy studies are needed to dictate the adequate technique to follow in the presence of IDP compared with what is currently described.

## DISCLOSURES

Author contributions: JS Frías-Ordoñez collected the data, reviewed the literature, drafted the manuscript, contributed to the interpretation of the data, and is the guarantor of the article. MAG Olarte revised the manuscript and contributed to the interpretation of the data. G. Hernández-Cely extensively revised the manuscript, performed patient management, and contributed to the interpretation of the data.

Financial disclosure: None to report.

Informed consent was obtained for this case report.
